# Monitoring tissue oxygenation index using near‐infrared spectroscopy during pre‐hospital resuscitation among out‐of‐hospital cardiac arrest patients: a pilot study

**DOI:** 10.1186/s13049-021-00857-7

**Published:** 2021-03-04

**Authors:** Jumpei Tsukuda, Shigeki Fujitani, Mahbubur Rahman, Kenichiro Morisawa, Takeshi Kawaguchi, Yasuhiko Taira

**Affiliations:** 1 Department of Emergency and Critical Care Medicine, St. Marianna University School of Medicine, 2-16-1 Sugao, Miyamae, 216-8511 Kawasaki, Kanagawa Japan; 2 Emergency Medicine, Thomas Jefferson University, 1020 Walnut Street, 19107 PA Philadelphia, USA; 3 Graduate School of Public Health, St. Luke’s International University, 3-6-2 Tsukiji, 104-0045 Tokyo, Japan

**Keywords:** Cardiopulmonary resuscitation, Out‐of‐hospital cardiac arrest

## Abstract

**Background:**

Tissue oxygenation index (TOI) using the near infrared spectroscopy (NIRS) has been demonstrated as a useful indicator to predict return of spontaneous circulation (ROSC) among out-of-hospital cardiac arrest (OHCA) patients in hospital setting. However, it has not been widely examined based on pre-hospital setting.

**Methods:**

In this prospective observational study, we measured TOI in pre-hospital setting among OHCA patients receiving cardio-pulmonary resuscitation (CPR) during ambulance transportation between 2017 and 2018. Throughout the pre-hospital CPR procedure, TOI was continuously measured. The study population was divided into two subgroups: ROSC group and non-ROSC group.

**Results:**

Of the 81 patients included in the final analysis, 26 achieved ROSC and 55 did not achieve ROSC. Patients in the ROSC group were significantly younger, had higher ∆TOI (changes in TOI) (5.8 % vs. 1.3 %; *p* < 0.01), and were more likely to have shockable rhythms and event witnessed than patients in the non-ROSC group. ∆TOI cut-off value of 5 % had highest sensitivity (65.4 %) and specificity (89.3 %) for ROSC. Patients with a cut-off value ≤-2.0 % did not achieve ROSC and while all OHCA patient with a cut-off value ≥ 8.0 % achieved ROSC. In addition, ROSC group had stronger positive correlation between mean chest compression rate and ∆TOI (*r* = 0.82) than non-ROSC group (*r* = 0.50).

**Conclusions:**

This study suggests that ∆ TOI could be a useful indicator to predict ROSC in a pre-hospital setting.

## Background

More than 100,000 people die from out-of-hospital cardiac arrest (OHCA) each year in Japan [1]. Although American Heart Association (AHA) guidelines for cardiopulmonary resuscitation (CPR) are regularly updated and the survival rate has been improved, the overall mortality of admitted OHCA patients still remain poor [2]. Survival rate after 1 month varied from 5.6 to 7.4 % regardless of the initial cardiac rhythm in Japan [1]. Brain is a vital organ with high metabolic activity and low energy storages and vulnerable to circulatory arrest [3, 4]. High-quality CPR according to the AHA guidelines for cardiopulmonary arrest (CPA) (with proper rate of 100–120 /min, proper depth of 5–6 cm, complete chest recoil and minimizing interruption of chest compressions measured by chest compression fraction and so on) can maintain cerebral blood flow only by 30–40 % of normal flow [5, 6]. Even if return of spontaneous circulation (ROSC) is obtained, brain injury remains as the leading cause of death after ROSC [7]. Some markers using neurological findings, imaging techniques and serum biomarkers, are known to evaluate the extent of brain injury and are only useful after ROSC [8]. However, there are no specific and reliable indicators to assess the cerebral blood flow directly to the response of CPR quality [9].

Near-infrared spectroscopy (NIRS) can provide information on oxygen saturation of brain tissue (StO_2_) non-invasively and continuously during CPR without a pulsating rhythm [10]. NIRS can measure StO_2_ from the ratio of oxygenated hemoglobin (O_2_Hb) to oxygenated and deoxygenated hemoglobin (HHb) in blood flow within venous, arterial and cerebral cortical tissue [7]. Several studies examined the correlation between StO_2_ and ROSC or neurological outcomes based on hospitalized patients [11, 12]. In our previous study which included 117 OHCA patients, we observed that ROSC patients had significantly higher initial StO_2_ than non-ROSC patients [13]. Other studies have also demonstrated that increase in StO_2_ (∆StO_2_) were associated with ROSC [12, 14]. In addition, usefulness of StO_2_ as a dynamic value rather than a single static value has also been emphasized [15]. Our previous study also showed that ∆StO_2_ could be more useful and accurate than a single initial StO_2_ when predicting ROSC [16]. Furthermore, in a recent meta-analysis, ∆StO_2_ demonstrated excellent predictive value for ROSC [15]. However, there is a dearth of well-designed studies which examined the association between high-quality CPR and level of StO_2_ during CPR, although a recent study based on small sample size demonstrated that high-quality CPR improved StO_2_ values [12].

In Japan, the average time from emergency medical service (EMS) call to hospital arrival was 39.4 minutes in 2016 and the time has been increasing every year [1]. Brain can reserve only limited energy, and inadequate cerebral blood flow within 5 minutes can lead to hypoxic brain injury [17]. For every minute without CPR and defibrillation, the chance of survival decreases by 7–10 % [18]. In order to improve the quality of pre-hospital CPR, evaluation of direct cerebral blood flow is necessary. Portable NIRS device equipped in ambulance, where only limited devices can be equipped, can help evaluate the cerebral blood perfusion [19, 20], especially oxygen delivery to the brain. Effective CPR might increase O_2_Hb and StO_2_ and through these mechanism ROSC rate can be improved. However, based on pre-hospital setting, the association between various types of StO_2_ and, ROSC and CPR according to the latest CPR guidelines has not been examined yet.

The objective of this study was to examine the association between ∆StO_2_ and ROSC as well as between ∆StO_2_ and the CPR quality.

## Methods

### Study design and setting

This single-center prospective and observational study was conducted at St. Marianna University School of Medicine, a 1200-bed tertiary hospital in Kawasaki, Japan. Enrollment for this study started from May 2017 and continued till March 2019. The research protocol received ethics committee Institutional Review Board (IRB) approval. In Japan, paramedic’s Advanced Life Support (ALS) team consists of three persons, under the direction of a physician while they are permitted to administer epinephrine and perform intubation. Additional devices are allowed to use only in the five EMS teams in northern Kawasaki medical area as all of these teams completed required training sessions before this study started.

### Study intervention

All ≥ 18 years old OHCA patients with non-traumatic cardiac arrest transferred to our emergency department (ED) were included. Excluded patients were as follows: patients with traumatic cause, patients with core body temperature less than 30°Celsius and patients who had achieved ROSC before the placement of the device probe. When the patients met inclusion criteria, the probe was placed onto the patient’s forehead left-laterally above the eyebrow immediately after transportation to the ambulance. One of the 3 paramedics placed the probe to minimize interruption of CPR procedure according to the AHA guidelines 2015 [21]. Although the paramedics were not blinded, they have not received the explanation about the meaning of the values, and followed the latest AHA guidelines without considering the StO_2_ values. They were instructed to administer epinephrine and perform tracheal intubation. They did not use mechanical chest compressions but did only manual chest compressions.

We used CCR-1^®^ (Hamamatsu Photonics, Hamamatsu-City, Shizuoka, Japan) which can non-invasively and continuously measures StO_2_, so called tissue oxygenation index (TOI) in CCR-1^®^. This device is portable and can be operated by battery for 2 hours which make it suitable for use in an ambulance. TOI monitoring continued throughout ambulance transportation. Initial ROSC was defined as the presence of a palpable carotid pulse after CPR discontinuation, and successful ROSC was defined as ROSC > 20 minutes after CPR [22].

### Measurements and statistical analysis

The study population was divided into 2 groups according to outcome: ROSC group and non-ROSC group. We defined initial TOI as the TOI measured at the moment the probe was attached inside the ambulance and final TOI as the last recorded TOI value at the arrival of the patient to our ED. We evaluated the change of TOI, namely the ∆TOI (∆TOI = final TOI - initial TOI). In addition to these values, mean, maximum and minimum TOI during ambulance transportation were also assessed. This device can also calculate the chest compression (CC) rate per minute using the waveform of O_2_Hb and HHb. Additional data were extracted from pre-hospital and hospital records according to Utstein style [23]. Pre-hospital records included the information about sex, age, the initial cardiac rhythm of cardiopulmonary arrest (CPA), witness, bystander CPR and the time from the EMS call to the scene arrival and hospital arrival. The hospital records included the causes of CPA, the amount of epinephrine received during CPR procedure, laboratory data and the outcomes in ED.

Primary aim of the statistical analysis was to examine the association between ∆TOI and ROSC. Secondary aims were to examine the association between other TOI values such as initial, final, mean, maximum and minimum and ROSC. In addition, different cut-off values of ∆TOI were examined as predictors of ROSC. We also examined the correlation between mean CC rate and ∆TOI using spearman’s rank correlation coefficient (r).

Continuous variables were summarized as median with interquartile range (IQR) or mean with standard deviation (SD). Distribution of continuous variables was examined using Shapiro-Wilk test. When the variables were normally distributed, unpaired t-tests were conducted. On the other hand, when the variables were positively or negatively skewed, we used Mann-Whitney U-tests. Categorical variables were summarized using counts and percentages, compared using chi-square test. Multiple regression analysis was conducted to examine the association between ∆TOI and Utstein variables after controlling for the potential confounding effects. Receiver Operating Characteristic (ROC) analysis was also conducted to determine the specific cut-off values predictive of ROSC. A p-value < 0.05 was considered statistically significant. Statistical analyses were performed using SPSS, version 25 (SPSS Inc., Chicago, IL, USA) and R statistical software (V.1.0.143, R Foundation for Statistical Computing).

## Results

**Fig. 1:**
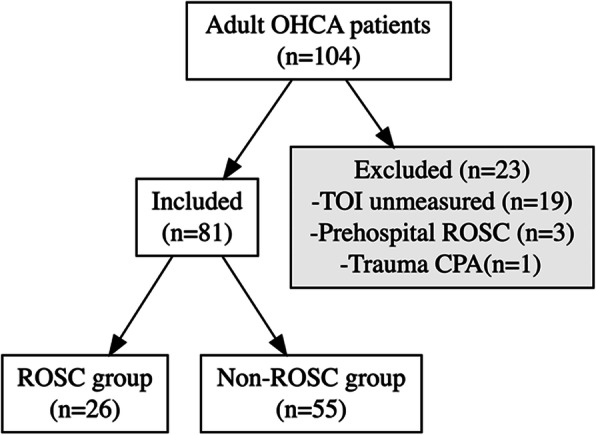
Flow chart of patient inclusion. Abbreviations: OHCA Out-of-hospital cardiac arrest, TOI Tissue oxygenation index, ROSC Return of spontaneous circulation, CPA Cardiopulmonary arrest.

Of 104 patients that were transported to our ED, TOI was measured in 81 (77.8 %) patients and 23 were excluded. The reasons for exclusion were; 19 patients were due to apparatus dysfunction during initial CPR procedure (i.e.: attachment failure of probes and start-up delay), 3 patients achieved ROSC before arrival at ED and one patient had CPA due to trauma. Among those who were included in this study (n = 81), 26 (32.1 %) achieved ROSC (ROSC group) and 55 (67.9 %) did not achieve ROSC (non-ROSC group) (Fig. 1).

Patients’ demographics and key characteristics according to Utstein style were compared between ROSC and non-ROSC group (Table 1). Patients in ROSC group were younger and were more likely to have their cardiac event witnessed. Furthermore, patients in this group exhibited higher shockable initial rhythm and suspected cardiac cause of CPA. Blood gas analysis showed that patients in ROSC group had significantly lower lactate concentration and higher PaO_2_ than patients in non-ROSC group.

**Table 1:** Patient characteristics

	ROSC group (*n* = 26)	Non-ROSC group (*n* = 55)	
Male, n (%)	19 (73.1)	32 (57.1)	0.166
Age (years), mean (SD)	72.4 (13.9)	80.8 (11.8)	0.006
Shockable rhythm, n (%)	7 (26.9)	1 (1.8)	< 0.01
Witness, n (%)	16 (61.5)	9 (16.4)	< 0.01
Bystander CPR, n (%)	10 (38.5)	24 (42.9)	0.707
Time from EMS call to the scene (min), median [IQR]	9 [8-10]	8 [6-10]	0.099
Time from EMS call to the hospital (min), median [IQR]	32 [27–36]	34 [29–40]	0.245
Time during ambulance (min), median [IQR]	8.5 [6-10]	9 [7-12]	0.089
Epinephrine dose during ambulance (mg), median [IQR]	1 [0–2]	1 [0–3]	0.48
Suspected cardiac cause, n (%)	11 (42.3)	3 (5.4)	< 0.01
Blood gas analysis	ROSC group	Non-ROSC group	
pH, mean (SD) ^a^ PaCO_2_ (Torr), median [IQR] ^a^ PaO_2_ (Torr), median [IQR] ^a^ HCO_3_^−^ (mmol/L), mean (SD) ^a^ Blood sugar (mg/dl), median [IQR] ^b^ Lactate (mmol/L), mean (SD) ^c^ Potassium (mmol/L), mean (SD) ^d^	6.94 (0.19) 65.3 [44.2–90.0] 96.2 [31.0-274.9] 13.8 (6.2) 265 [209–388] 9.7 (4.6) 5.5 (1.5)	6.75 (0.22) 90.5 [67.4122.3] 38.7 [22.5–77.9] 12.7 (5.2) 173 [102–268] 13.4 (4.4) 7.2 (1.7)	< 0.01 0.012 0.007 0.427 0.013 0.002 < 0.01

^a^The number of non-ROSC group was 44

^b^The number of non-ROSC group was 33

^c^The number of non-ROSC group was 38

^d^The number of non-ROSC group was 30

Abbreviations: *CPA* cardiopulmonary arrest, *CPR* cardiopulmonary resuscitation, *EMS* emergency medical services, *ROSC* return of spontaneous circulation, *TOI* tissue oxygenation index

### Primary outcome measurement

**Fig. 2:**
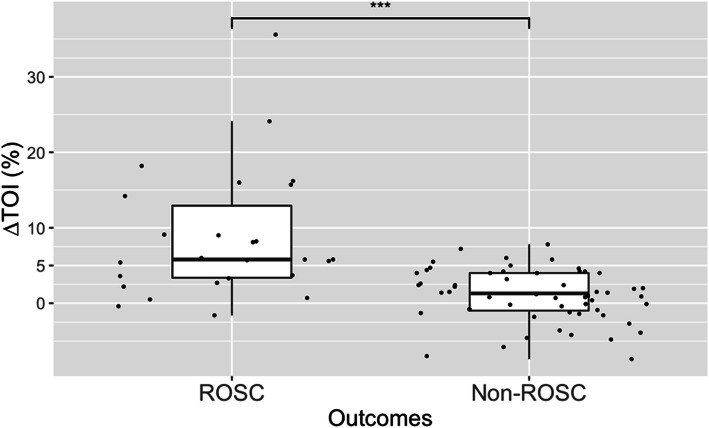
Distributions of ∆TOI for patients with out-of-hospital cardiac arrest (OHCA) by ROSC status***: *p* < 0.01 . Abbreviations: ROSC Return of spontaneous circulation, TOI Tissue oxygenation index

∆TOI was significantly higher in ROSC group (median 5.8 % [IQR3.2 to14.6 %]) than non-ROSC group (median 1.3 % [IQR-1.1 to -1.3 %]) (*p* < 0.01) (Fig. 2).

### Secondary outcome measurement

Initial, final, minimum, maximum and mean TOI values were also significantly higher in ROSC group than that in non-ROSC group (Table 2).

**Table 2:** Various type of tissue oxygenation index (TOI) and outcomes

	ROSC group (*n* = 26)	Non-ROSC group (*n* = 55)	*p*-value
ΔTOI (%), median [IQR]	5.8 [3.2–14.6]	1.3 [-1.1-1.3]	< 0.01
Initial TOI (%), mean (SD)	33.6 (8.5)	29.6 (7.6)	0.034
Final TOI (%), mean (SD)	42.2 (10.4)	30.6 (8.2)	< 0.01
Maximum TOI (%), mean (SD)	52.8 (14.0)	42.8 (10.5)	< 0.01
Minimum TOI (%), mean (SD)	26.8 (9.2)	19.2 (9.6)	< 0.01
Mean TOI (%), mean (SD)	37.9 (9.0)	30.3 (8.0)	< 0.01

Abbreviations: *TOI* tissue oxygenation index, *ROSC* return of spontaneous circulation

Table 3 shows crude and adjusted odds ratio of achieving ROSC based on logistic regression analysis. Among different TOI values, ∆TOI had highest odds ratio for predicting ROSC based on bivariate logistic regression analysis. Even after adjusted by witness status and shockable rhythm, the association between ∆TOI and ROSC was statistically significant, although other TOI values were not. (Table 3).

**Table 3:** Odd ratio of ROSC prediction for each TOI and ∆ TOI after adjustment for baseline variables

	Crude OR (95 % CI)	Adjusted OR ratio (95 % CI)
∆ TOI	1.42 (1.18–1.71)	
Initial TOI	1.07 (1.00-1.15)	
Final TOI	1.21 (1.10–1.34)	
Maximum TOI	1.08 (1.03–1.13)	
Minimal TOI	1.09 (1.03–1.16)	
Mean TOI	1.17 (1.03–1.29)	
Adjusted-∆TOI		1.46 (1.16–1.8)

Adjusted for shockable rhythm and witness which were best 2 predictive indicators for ROSC

Abbreviations: *ROSC* return of spontaneous circulation, *TOI* tissue oxygenation index, *OR* odds ratio

**Fig. 3:**
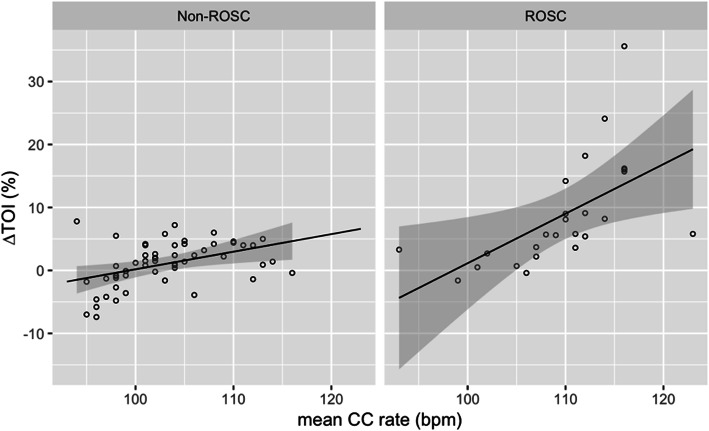
Correlation between ∆TOI and mean CC rate The shaded region indicated 95 % CI. Abbreviations: ROSC Return of spontaneous circulation, TOI Tissue oxygenation index, CC Chest compression, CI Confident interval

Figure 3 shows correlation between CC rate and ∆TOI during ambulance transportation. Overall, there was statistically significant positive correlation between CC rate and ∆TOI (*r* = 0.65). ROSC group had stronger positive correlation between CC rate and ∆TOI (*r* = 0.82) than non-ROSC group (*r* = 0.50).

**Fig. 4:**
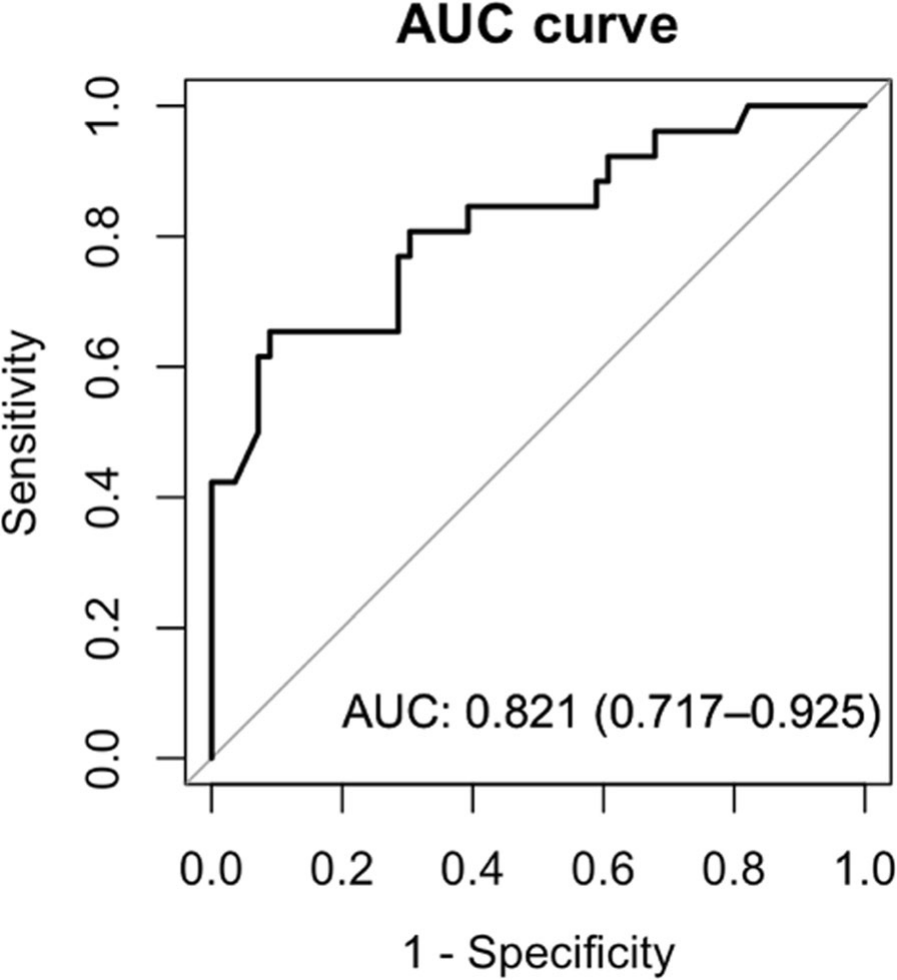
ROC curve with ∆TOI as a predictor of ROSC. Abbreviation: AUC Area under the curve

**Fig. 5:**
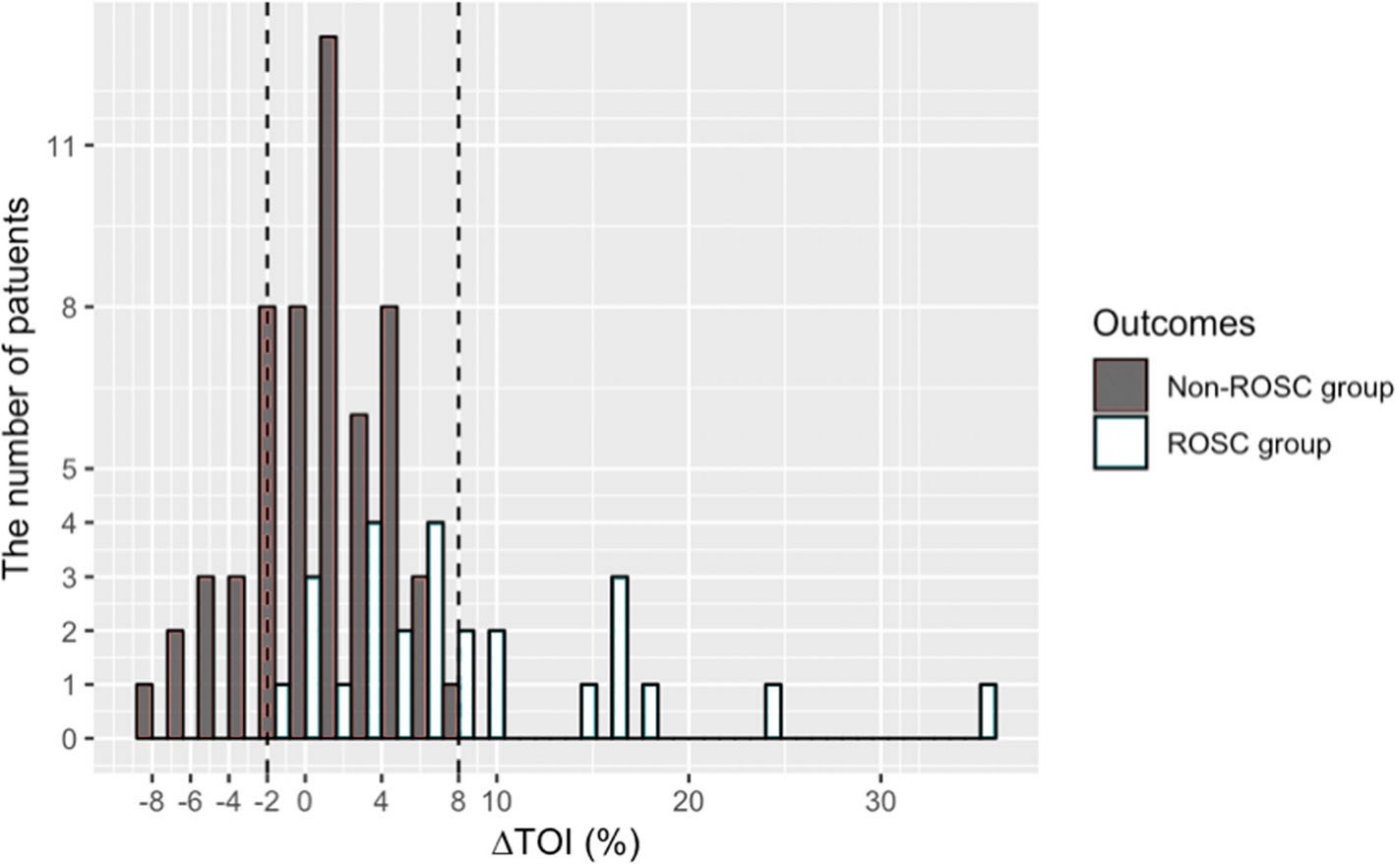
∆ tissue oxygenation index (TOI) of each patient. Abbreviation: ROSC Return of spontaneous circulation

ROC analysis showed that ∆TOI cut-off value 5 % had the highest sensitivity and specificity to predict ROSC (65.4 and 89.3 %, respectively). The area under the ROC curve (AUC) was 0.82 (95 % confidence interval, 0.72–0.93) (Fig. 4). Patients with OHCA whose ∆TOI was ≤-2.0 % did not achieve ROSC, whereas patients with OHCA whose ∆TOI was ≥ 8.0 % achieved ROSC (Fig. 5).

## Discussion

To the best of our knowledge, this is the first study conducted in Japan which demonstrated that ∆TOI is a significant predictor of ROSC even after adjusting for Utstein variables in a pre-hospital setting. Several other studies also reported regional cerebral oxygen saturation (rSO_2_) as a correlate of ROSC status in a hospital setting [24]. Together, these studies emphasize that initial rSO_2_ as well as increase in rSO_2_ (∆rSO_2_) could be regarded as a useful parameter to assess ROSC in hospital and pre-hospital settings [19, 20].

Utstein variables are widely used to determine the predictive indicators associated with ROSC [23]. Among these variables, initial cardiac rhythm, witness, bystander CPR, time from EMS call to scene arrival and cardiac cause are especially known as core Utstein variables [24]. Similar to our previous study based on hospital setting, we also observed in this study that adding witness status and initial shockable rhythm to ∆TOI in pre-hospital setting increased the accuracy of ROSC prediction [13]. Other study with larger sample size also demonstrated that witness and shockable rhythm had significant association with ROSC [20]. We also observed stronger correlation between CC rate and ∆TOI among patients in the ROSC group than in non-ROSC group. Thus, ∆TOI might also be considered as an indicator of high-quality CPR in addition to its effectiveness as a ROSC predictor.

Determining the cut-off values might suggest that TOI could potentially replace pulse checks during CPR, which could reduce hands-off time. TOI increases when CPR delivers O_2_Hb to the brain. ∆TOI as a dynamic value might reflect the quality of CPR. ∆rSO_2_ ≥ 15 % during CPR procedure showed higher chance of achieving ROSC in a previous study[20]. In our study, we observed that ∆TOI cut-off value 5.0 % could predict ROSC. This discrepancy was due to difference in using the parameter as predictors in the respective study (∆rSO_2_ v.s. ∆TOI) as well as differences in the calculation method[13]. Different cut-off values of ∆TOI generated in this study to predict the probability of ROSC (∆TOI ≤-2 % did not achieve ROSC ≥ 8 % achieved ROSC) are study-specific values only. Although these values are based on the findings of our study, future studies might shed more light on appropriate cut-off values.

CCR-1^®^ can measure mean CC rate from the waveform of O_2_Hb and HHb. ∆TOI and CC rate showed significant positive correlation in this study with stronger correlation in ROSC group than non-ROSC group. Appropriate CC rate based on CPR guidelines is 100–120 per minute [5]. Surprisingly, 23 out of 81 people (28.4 %) could not comply with the latest CPR guidelines in this study. As in the narrow space of the ambulance, chest compressions are not always performed according to the guidelines, visual NIRS monitoring might replace the evaluation of CPR quality.

∆TOI as a dynamic value was more specific indicator to predict ROSC than other static TOI values. These results are similar to other studies based on NIRS monitoring in pre-hospital setting [19, 20]. TOI is expressed as the ratio of O_2_Hb and HHb, and it increases with O_2_Hb level. Blood gas analysis showed that ROSC group had higher PaO_2_ and lower PaCO_2_ than non-ROSC group. Experimental animal CPR model also showed that rSO_2_ was lower with 50 % oxygen than 100 % oxygen [25]. Together, these studies support the use of TOI as a dynamic value to predict ROSC in both pre-hospital and hospital settings.

Our study has several limitations. This study was conducted in a single center, and the sample size was small because only 5 EMS teams could be equipped with portable NIRS device. There were many date errors attached to probe performance. Also, we had only a few subjects with neurological event, therefore we could not evaluate the association between TOI values and neurological outcomes. Portable NIRS device could not measure the depth of CC during CPR and we could only evaluate the correlation between mean CC rate and ∆TOI. Finally, we did not have laboratory data and, therefore, could not evaluate the change in PaO_2_ during ambulance transportation.

## Conclusions

In this pilot study, we demonstrated the feasibility of ∆TOI as a dynamic value rather than single static value among OHCA patients in a pre-hospital setting. ∆TOI can be considered as a predictor of ROSC and can guide CC rate. Other findings, such as, an absolute increase of 8 % or higher in TOI during pre-hospital CPR procedure is associated with ROSC and absolute decrease of 2 % or lower from the baseline is associated with non-ROSC, would be helpful to generate future cut-off values in this regard.

## Data Availability

The datasets used and/or analyzed during the current study are available from the corresponding author on reasonable request.

## Abbreviations

TOI: Tissue oxygenation index
NIRS: Near infrared spectroscopy
ROSC: Return of spontaneous circulation
OHCA: Out-of-hospital cardiac arrest
CPR: Cardio-pulmonary resuscitation
AHA: American Heart Association
CPA: Cardiopulmonary arrest
StO_2_: Oxygen saturation of brain tissue
O_2_Hb: Oxygenated hemoglobin
HHb: Deoxygenated hemoglobin
∆StO_2_: Increase in StO_2_
EMS: Emergency medical service
IRB: Institutional Review Board
ALS: Advanced Life Support
ED: Emergency department
TOI: Tissue oxygenation index
CC: Chest compression
IQR: Interquartile range
SD: Standard deviation
ROC: Receiver Operating Characteristic
rSO2: Regional cerebral oxygen saturation
